# Financial disincentives? A three-armed randomised controlled trial of the effect of financial Incentives in Diabetic Eye Assessment by Screening (IDEAS) trial

**DOI:** 10.1136/bjophthalmol-2017-311778

**Published:** 2018-05-23

**Authors:** Gaby Judah, Ara Darzi, Ivo Vlaev, Laura Gunn, Derek King, Dominic King, Jonathan Valabhji, Colin Bicknell

**Affiliations:** 1 Department of Surgery and Cancer, Imperial College London, London, UK; 2 Warwick Business School, University of Warwick, Coventry, UK; 3 Public Health Program, Stetson University, DeLand, Florida, USA; 4 Personal Social Services Research Unit (PSSRU), London School of Economics and Political Science, London, UK; 5 Department of Medicine, Imperial College London, London, UK

**Keywords:** Public health, Retina

## Abstract

**Objective:**

Conflicting evidence exists regarding the impact of financial incentives on encouraging attendance at medical screening appointments. The primary aim was to determine whether financial incentives increase attendance at diabetic eye screening in persistent non-attenders.

**Methods and analysis:**

A three-armed randomised controlled trial was conducted in London in 2015. 1051 participants aged over 16 years, who had not attended eye screening appointments for 2 years or more, were randomised (1.4:1:1 randomisation ratio) to receive the usual invitation letter (control), an offer of £10 cash for attending screening (fixed incentive) or a 1 in 100 chance of winning £1000 (lottery incentive) if they attend. The primary outcome was the proportion of invitees attending screening, and a comparative analysis was performed to assess group differences. Pairwise comparisons of attendance rates were performed, using a conservative Bonferroni correction for independent comparisons.

**Results:**

34/435 (7.8%) of control, 17/312 (5.5%) of fixed incentive and 10/304 (3.3%) of lottery incentive groups attended. Participants who received any incentive were significantly less likely to attend their appointment compared with controls (risk ratio (RR)=0.56; 95% CI 0.34 to 0.92). Those in the probabilistic incentive group (RR=0.42; 95% CI 0.18 to 0.98), but not the fixed incentive group (RR=1.66; 95% CI 0.65 to 4.21), were significantly less likely to attend than those in the control group.

**Conclusion:**

Financial incentives, particularly lottery-based incentives, attract fewer patients to diabetic eye screening than standard invites in this population. Financial incentives should not be used to promote screening unless tested in context, as they may negatively affect attendance rates.

## Introduction

Diabetes and its complications costs the National Health Service (NHS) over £10 billion annually, representing 10% of the NHS budget for England and Wales.^1^ These costs exclude indirect costs to the UK economy, including absenteeism, early retirement and social benefits. Costs are expected to rise given trends of rising diabetes prevalence.

Diabetic retinopathy is a leading cause of sight loss in the UK working population^2^ and is associated with 1280 new cases of blindness annually.^3^ The diabetic eye screening (DES) programme identifies early stages of retinopathy, before symptoms develop and sight loss occurs, to enable effective treatment. The uptake rate for DES is 83% (2015–2016).^4^ Therefore, many with diabetes are not screened and at risk of avoidable sight loss.

While screening programmes such as DES are beneficial overall, they can potentially exacerbate health inequalities. In the UK, diabetes prevalence increases with increasing deprivation, while diabetic retinopathy screening attendance decreases and the prevalence of sight-threatening retinopathy among screened patients increases.^5^ Cost-effective strategies are required to increase screening uptake, particularly in those most at risk.

One strategy gaining popularity is financial incentives to encourage healthy behaviours.^6^ There are many examples where financial incentives have significantly changed behaviour at a relatively low cost. However, applying principles from behavioural economics can increase effectiveness, as the incentive structure can affect their impact.^7^ Financial incentives may also be more effective at influencing infrequent health behaviours, such as vaccination uptake, rather than producing sustained behaviour change, for example, smoking cessation.^8^ Therefore, incentives may be an effective and cost-effective strategy to promote screening uptake. Financial incentives have not previously been investigated in a randomised trial of DES uptake.

The primary objective was to test whether financial incentives increase attendance in persistent non-attenders. Secondary objectives included whether the design of the incentive scheme influences its effectiveness and whether patients attending DES from the incentive groups were from a different sociodemographic background than the control group.

## Methods

This study was registered with the International Standard Randomised Controlled Trial Number registry (ISRCTN14896403). The report for the funder (National Institute for Health Research) is available online,^9^ and justification of the intervention conditions can be found in the published protocol.^10^

### Trial design

The Incentives in Diabetic Eye Assessment by Screening (IDEAS) trial was a three-arm randomised controlled trial, with a 1.4:1:1 ratio between the control, fixed incentive and lottery incentive groups, respectively. Participants were sent a standard appointment letter inviting them to attend a DES appointment at a fixed time.

### Participants

Eligible participants were diabetic patients, aged 16 years or over, from general practitioner (GP) practices in Kensington, Chelsea and Westminster, who had been invited to annual eye screening in the last 24 months but had failed to attend or rearrange a subsequent appointment.

Participants were identified from the 1st Retinal Screening Ltd database, a contracted company responsible for screening in the area, on the 12 March 2015. The normal, annual invitation process continued for trial participants. A minimum period of 2 months was left between appointment letters sent as usual care and appointment letters for the trial to ensure that no patient was enrolled who was late to contact the screening service but intended to do so. Before letters were sent, participant details were checked against the GP patient register to check address details and so that patients who had become ineligible were not invited.

### Procedure

Eligible participants were sent a standard appointment invitation letter 4 weeks before their appointment, with or without incentive (according to randomisation). Clinic dates were alternated between the three conditions, (interspersed with extra control clinics given the larger group size) to control for any seasonal variability in attendance. Participants could reschedule this appointment once and remain eligible for the incentive.

Dedicated IDEAS clinics were arranged within St Mary’s hospital, London, (between 19 March and 20 August 2015) separately for each trial condition to ensure participant blinding to the different incentive conditions. At the end of the study, anonymised demographic and screening attendance data were collected from the central database.

### Interventions

The design of the financial incentives took into account established psychological phenomenon from Kahneman’s Prospect Theory.^11^ The incentive vouchers included expiry dates (the screening date) to introduce an aspect of ‘loss aversion’, whereby losing a reward is more powerful than gaining a reward.^12^

#### Control

Participants received a standard letter from the screening service, inviting them to an appointment on a fixed date and time. A telephone number was provided for patients to rearrange their appointment if necessary.

#### Fixed incentive

**Figure 1:**
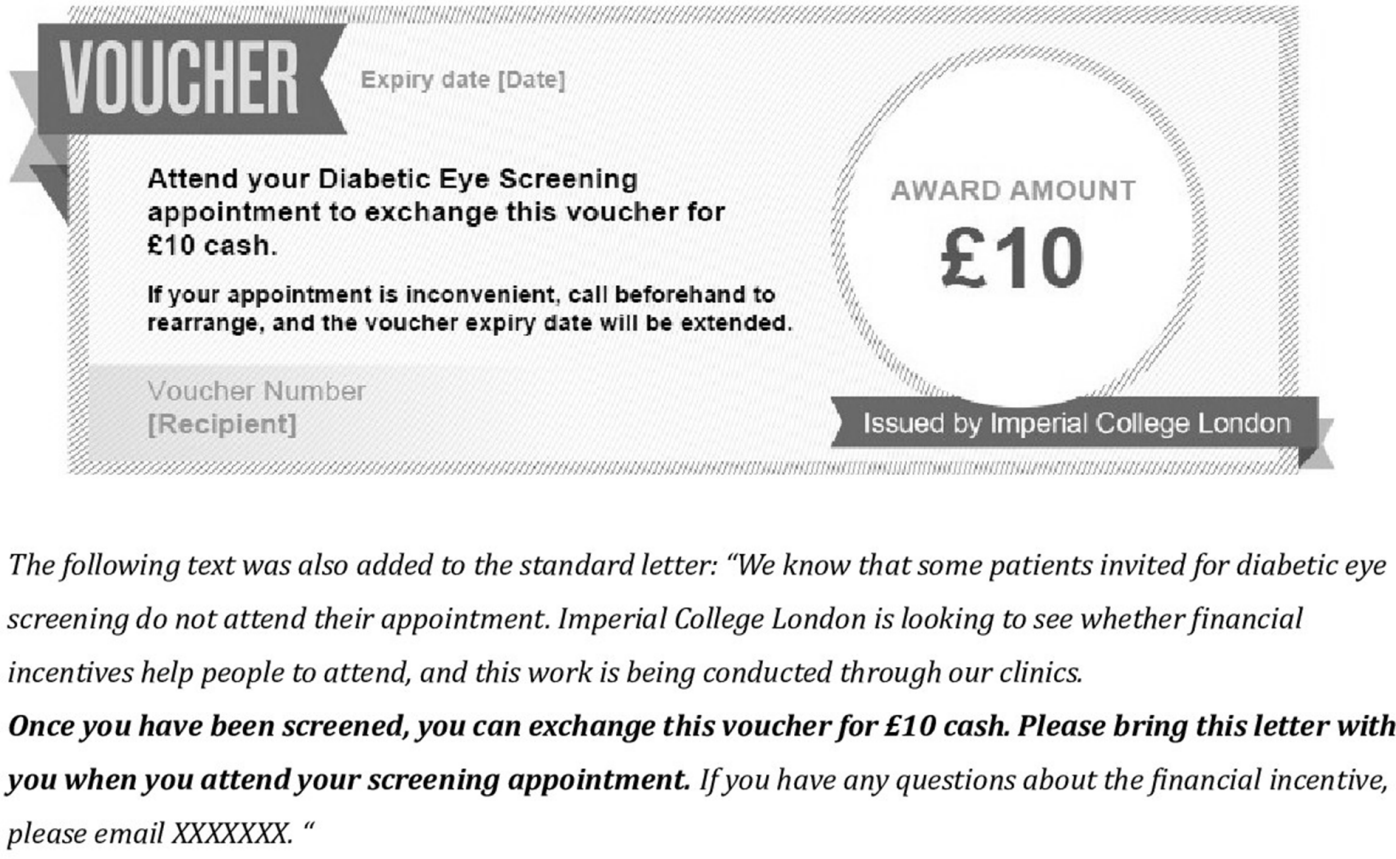
Image of voucher and supplementary text added to the invitation letter for the fixed incentive group.

Participants received the standard invitation letter as in the control group, with the inclusion of additional text and a voucher offering a financial incentive of £10 for attending screening (figure 1). £10 was chosen considering the concept of reference points—offering a small incentive encourages people to collect test results, but increasing the value has little effect.^13^ £10 was estimated to cover patients’ time or travel cost.

#### Lottery incentive

**Figure 2:**
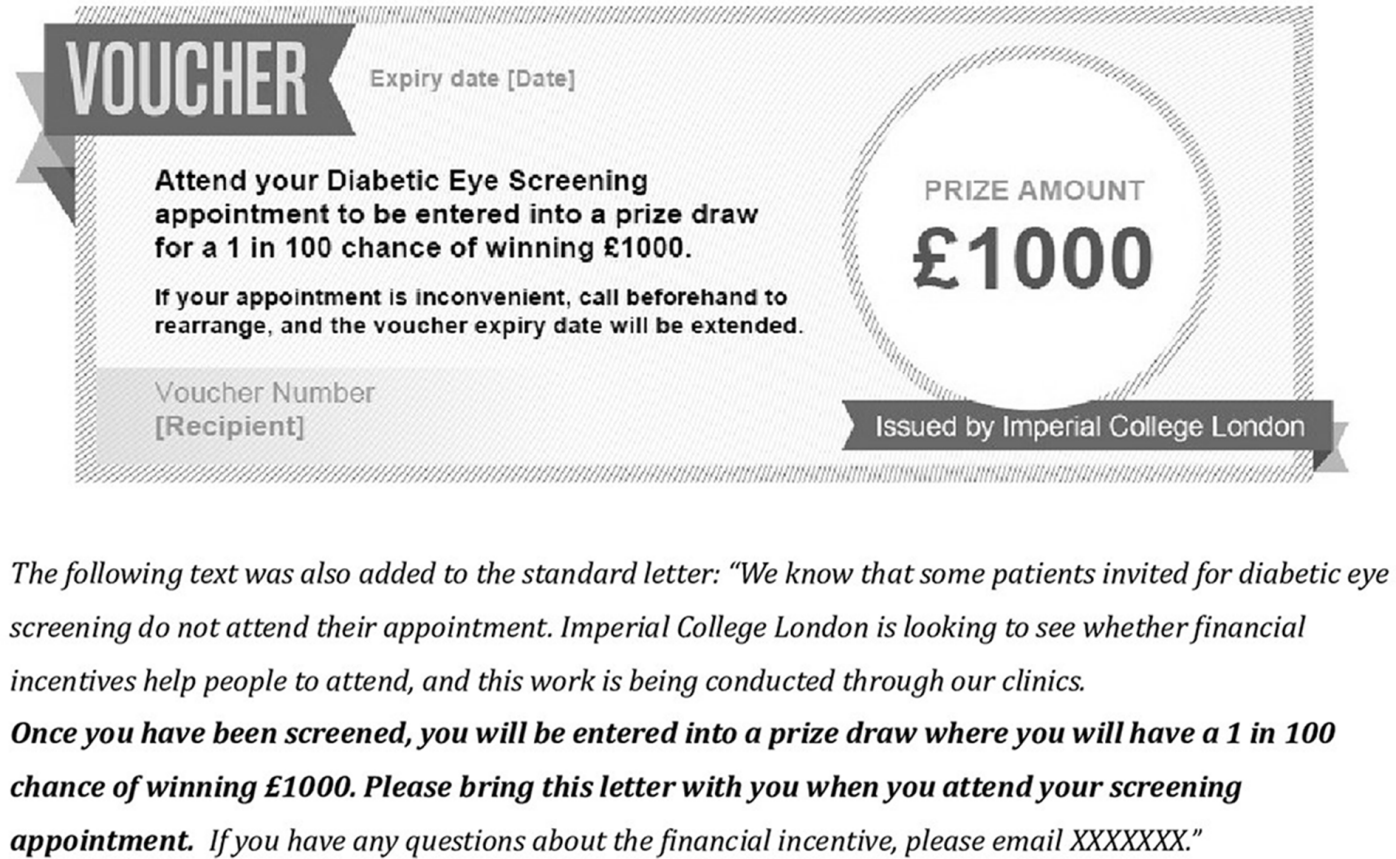
Image of voucher and supplementary text added to the invitation letter for the lottery incentive group.

Participants received the standard invitation letter, including additional text and a voucher offering entry into a prize draw with a 1 in 100 chance of winning £1000 following screening (figure 2). This condition was designed considering the phenomena that people overvalue small probabilities, explaining the popularity of lotteries and insurance.^11^ Given limited resources in an incentive programme, offering a chance of winning a lottery may be more effective than smaller individual rewards.^14^ The lottery probability was chosen so that it would be easy to comprehend,^7^ and the value was chosen so that the ‘expected value’ was equivalent for both incentive conditions (100% chance of winning £10 versus a 1% chance of winning £1000).

### Outcomes

The primary outcome was screening attendance. Secondary outcome measures included the demographic profile of participants attending and not attending their appointment. Data were available on: Indices of Multiple Deprivation^15^ (IMD) (a measure of deprivation level based on small geographic areas, obtained through postcode data); straight line distance from the address to the screening centre; and number of years registered with the service. Data were presented as deciles for age and deprivation to maintain patient confidentiality.

The follow-up action recommended from screening, aggregated by treatment group, was a further outcome. This was converted into a binary score of ‘no additional management required’ versus ‘additional management required’.

### Randomisation and blinding

To generate the random allocation sequence, the data manager at 1st Retinal Screen Ltd provided the team statistician with a spreadsheet containing deidentified/anonymised patient data. For 1274 eligible patients provided in the list, participants were indexed according to numbers generated at random using a standard random number generator, with double precision, to avoid duplicates. Participants were then sorted from smallest to largest according to this random index. Within the sorted list and using the 1.4:1:1 randomisation ratio, the team statistician assigned: (1) the lowest 375 participants to the fixed incentive group; (2) the following 375 participants to the probabilistic incentive group; and (3) the remainder of the participants (ie, 524) to the control group. Due to this method of anonymised patient identifiers being allocated to groups by the trial statistician prior to the start of the study, there was no issue resulting from lack of concealment of the intervention sequence.

Given two intervention groups, each compared with a control, maximum statistical efficiency is achieved using a 1.4:1:1 randomisation ratio.^16^

Given the designated clinics for each trial condition, it was not possible for the researcher (who was present at intervention clinics to administer the incentive) or the screener to be blinded to group assignment. However, as screening attendance is the primary outcome, the results could not be biased by the lack of blinding at this stage.

### Sample size

As a group of persistent non-attenders, attendance in the control group was expected to be extremely low at 1%, with a 10% increase in attendance considered clinically significant. This was deemed feasible given other financial incentive studies, where smoking cessation rates among employees increased from 5% to 14.7%,^17^ and warfarin adherence improved from 65% to 97.8%.^18^ Given the primary analysis of a single test comparing the control group to the combined incentive groups, and assuming a 10% increase in attendance with the intervention, a total sample size of approximately 350 would achieve at least 95% power. The study was also powered for the secondary analysis of investigating whether there are differences in attendance given the different incentive conditions. To detect the same minimal clinically important difference of 10% (attendance increasing from 1% in the control group to 11% in an intervention group) with at least 95% power and a Bonferroni correction for the multiple comparisons in the secondary analysis, there would be 95% power with 1000 participants (412 in the control group and 294 in each intervention group). Should attendance in the control group be 5%, the study would still have approximately 85% power to detect a 10% increase in attendance to 15%.

### Analysis

Differences in demographic profile between randomised groups were calculated using χ^2^ analysis. For the primary analysis, a comparative analysis was performed, comparing attendance in the control group to the fixed and lottery incentive groups combined.

For secondary analyses, pairwise comparisons of attendance rates (absolute risk differences) were performed between control versus fixed incentive, control versus lottery incentive and fixed versus lottery incentive groups, adjusting for multiple comparisons using a Bonferroni correction.

The sociodemographic characteristics were compared between trial attenders and non-attenders, as well as attenders from control and incentive groups. Binary categories were used for age (above or below 65 years) and IMD (0–20 vs 30–60) when assessing differences by treatment group.

Group differences in the proportion of patients attending who require additional management following screening was assessed using χ^2^ analysis.

To explore whether incentive schemes attract patients with a different socioeconomic or demographic status, a backward stepwise multivariate logistic regression was performed to adjust the treatment effect by the demographic covariates. The statistical analysis plan (prepared in advance of the trial analysis) is shown as supplementary material.

## Results

**Figure 3:**
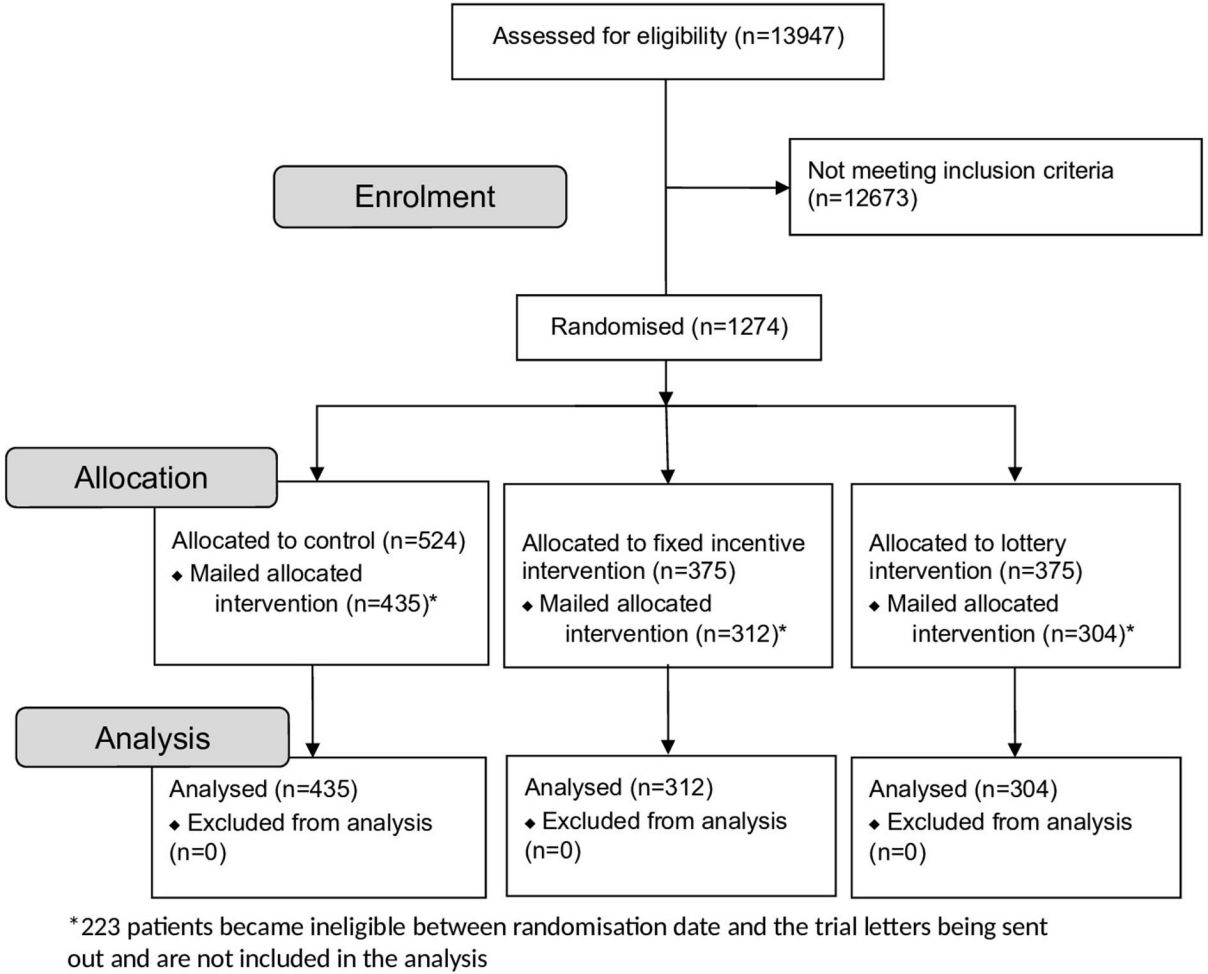
Trial participant flow diagram.

One thousand two hundred seventy-four patients were eligible and randomised. Between the randomisation date and patients being sent invitation letters, 223 became ineligible. Therefore, 1051 participants were sent an invitation letter and included in the analysis (n*=*435 control; n=312 fixed; n*=*304 lottery). The trial flow diagram is shown in figure 3. No harms occurred.

The primary reasons for ineligibility before being sent an appointment invitation letter were: attendance at annual DES appointment (44.4% of ineligible patients) and moving out of the area (22.4%). The loss of patients did not affect the sample size target or predefined ratio. There were no group differences in reasons for ineligibility (p=0.736). The demographic profile of participants is shown in table 1.

**Table 1:** Demographics of patients included in the trial

	Overall invited IDEAS participants (n=1051)	Control(n=435)	Fixed(n=312)	Lottery(n=304)
Gender, n (%)				
Male	609 (57.9)	263 (60.5)	170 (54.5)	176 (57.9)
Female	442 (42.1)	172 (39.5)	142 (45.5)	128 (42.1)
Age (years), n (%)				
16–25	12 (1.1)	4 (0.9)	2 (0.6)	6 (2.0)
26–35	37 (3.5)	16 (3.7)	10 (3.2)	11 (3.6)
36–45	113 (10.8)	50 (11.5)	27 (8.7)	36 (11.9)
46–55	181 (17.2)	72 (16.6)	53 (17.0)	56 (18.4)
56–65	235 (22.4)	87 (20.0)	81 (26.0)	67 (22.0)
66–75	237 (22.5)	106 (24.4)	68 (21.8)	63 (20.7)
76–85	173 (16.5)	72 (16.5)	54 (17.3)	47 (15.5)
≥86	63 (6.0)	28 (6.4)	17 (5.4)	18 (5.9)
IMD decile, n (%)				
Most deprived 0≤IMD<10	29 (2.8)	7 (1.6)	9 (2.9)	13 (4.3)
10≤IMD<20	275 (26.2)	127 (29.2)	65 (20.8)	83 (27.3)
20≤IMD<30	252 (24.0)	100 (23.0)	85 (27.2)	67 (22.0)
30≤IMD<40	216 (20.5)	87 (20.0)	68 (21.8)	61 (20.1)
40≤IMD<50	158 (15.0)	66 (15.2)	51 (16.4)	41 (13.5)
50≤IMD<60	107 (10.2)	43 (9.9)	28 (9.0)	36 (11.8)
60≤IMD<70	14 (1.3)	5 (1.1)	6 (1.9)	3 (1.0)
Distance from clinic (km)				
Mean (SD)	2.7 (1.81)	2.69 (1.82)	2.81 (1.84)	2.59 (1.79)
Years registered on screening programme				
Mean (SD)	6.0 (2.17)	6.0 (2.12)	5.8 (2.23)	6.0 (2.20)

IDEAS, Incentives in Diabetic Eye Assessment by Screening; IMD, Index of Multiple Deprivation.

Sixty-one participants attended screening (5.8% of those invited). Thirty-four participants (7.8%) attended from the control arm and 27 participants (4.4%) from the two incentive arms combined. Table 2 shows pairwise comparisons of attendance rate by treatment group. Those in an incentive group were 44% less likely to attend screening compared with controls (RR=0.56; 95% CI 0.34 to 0.92).

**Table 2:** Pairwise comparisons of attendance rate by treatment group

Pairwise comparison	Risk difference(95% CI)	Relative risk(95% CI)
Any incentive versus control(primary aim with a single comparison)	−0.03 (−0.06 to 0.01)* p=0.02*	0.56 (0.34 to 0.92)* p=0.03*
Fixed incentive versus control(secondary aim using the Bonferroni adjustment for multiple comparisons)	−0.02 (−0.07 to 0.02) p=0.19	0.70 (0.35 to 1.39) p=0.26
Lottery incentive versus control(secondary aim using the Bonferroni adjustment for multiple comparisons)	−0.05 (−0.09 to 0.003)† p=0.01†	0.42 (0.18 to 0.98)† p=0.02†
Fixed versus lottery incentive(secondary aim using the Bonferroni adjustment for multiple comparisons)	0.02 (−0.02 to 0.06) p=0.19	1.66 (0.65 to 4.21) p=0.27

*Significant at the 0.05 significance level, as a single comparison.

†Significant at the 0.05 significance level, with a Bonferroni multiple comparison adjustment for three comparisons.

Seventeen participants (5.5%) attended from the fixed incentive arm, and 10 participants (3.3%) from the lottery arm. Those in the lottery arm were 58% less likely to attend screening compared with control participants (RR=0.42; 95% CI 0.18 to 0.98).

The sociodemographic characteristics of trial attenders and non-attenders for each group is shown in table 3. There were no significant differences in sociodemographic characteristics between attenders and non-attenders. No significant differences were found among trial attenders between the control and incentive groups. There was no difference between incentive and control groups in terms of outcome recommendation from screening (p=0.387). Multivariate analysis to determine the covariate-adjusted differences in attendance rates between groups demonstrated that none of the sociodemographic factors impacted on attendance.

**Table 3:** Sociodemographic profile of attenders and non-attenders in the IDEAS trial

	IDEAS participants (invited) (n=1051)	Trial non-attenders(n=990)	Trial attenders (n=61)				
			Total (n=61)	Control (n=34)	Incentive (n=27)		
					Total (n=27)	Fixed (n=17)	Lottery (n=10)
Gender							
Male, n (%)	609 (57.9)	577 (58.3)	32 (52.5)	20 (58.8)	12 (44.4)	9 (52.9)	3 (30.0)
Female, n (%)	442 (42.1)	413 (41.7)	29 (47.5)	14 (41.2)	15 (55.6)	8 (47.1)	7 (70.0)
		p=0.371Non-attenders versus attenders		p=0.264Incentive versus control			
Age (years)							
≤65, n (%)	578 (55.0)	541 (54.6)	37 (60.7)	19 (55.9)	18 (66.7)	10 (58.8)	8 (80.0)
>65, n (%)	473 (45.0)	449 (45.4)	24 (39.3)	15 (44.1)	9 (33.3)	7 (41.2)	2 (20.0)
		p=0.360Non-attenders versus attenders		p=0.392Incentive versus control			
IMD decile							
0–20, n (%)	556 (52.9)	522 (52.7)	34 (55.7)	19 (55.9)	15 (55.5)	10 (58.8)	5 (50.0)
30–60, n (%)	495 (47.1)	468 (47.3)	27 (44.3)	15 (44.1)	12 (44.4)	7 (41.2)	5 (50.0)
		p=0.648Non-attenders versus attenders		p=0.980Incentive versus control			
Distance from clinic (km)							
Mean (SD) median range	2.7 (1.81)2.50.0–17.5	2.71 (1.80)2.50.0–17.5	2.53 (1.94)2.00.5–10.0	2.94 (2.25)2.50.5–10.0	2.0 (1.32)2.00.5–6.5	2.09 (1.47)1.50.5–6.5	1.85 (1.06)2.00.5–3.5
		p=0.447;IMD=−0.1895% CI (−0.65 to 0.29)Non-attenders versus attenders		p=0.059;IMD=0.9495% CI (−0.04 to 1.92)Incentive versus control			
Years registered							
Mean (SD) median range	5.96 (2.17)7.02–8	5.96 (2.19)7.02–8	5.84 (1.95)6.02–8	5.79 (1.92)6.02–8	5.89 (2.03)6.02–8	5.65 (2.18)5.02–8	6.30 (1.77)6.53–8
		p=0.654;IMD=−0.1395% CI (−0.69 to 0.43)Non-attenders versus attenders		p=0.852;IMD=−0.9595% CI (−1.11 to 0.92)Incentive versus control			

IDEAS, Incentives in Diabetic Eye Assessment by Screening; IMD, Indices of Multiple Deprivation.

## Discussion

In this randomised controlled trial, financial incentives were not effective at improving uptake at DES clinics targeting persistent non-attenders. Contrary to expectations based on the theoretical application of behavioural economics, those who received an incentive offer were significantly less likely to attend screening than those who received a standard appointment invitation only. In addition, we found no evidence that the sociodemographic profile or screening outcome of patients attending following an incentive offer was any different to those attending following the standard appointment invitation.

While it was observed that those in an intervention group were statistically significantly less likely to attend than those in the control group, it should be noted that the effect size was small and not clinically significant, and this finding may not be generalisable due to the high ethnic diversity of the study population. Therefore, while there is strong evidence that the incentive did not increase DES attendance, it may not be correct to conclude that financial incentives would be expected to significantly reduce uptake in other contexts.

Participants were well matched between groups in terms of demographic factors; however, participants were from the most deprived and hard-to-reach populations overall. A percentage of 52.9 of participants were in the bottom three IMD deciles. These patients with diabetes were consistently not attending despite 60% of trial participants being registered for at least 6 years in the Kensington, Chelsea and Westminster DES programme. It is of great importance that this high-risk, non-attending group is screened. Previous studies in deprived populations have shown financial incentives to be particularly effective.^19^ In this trial, which included deprived participants, incentives were ineffective at increasing retinopathy screening participation and the incentive offer was associated with *lower* screening uptake, especially in the lottery group.

Patients with diabetes often feel well and may only be driven to attend health appointments after significant health events. It is possible that the target population have not experienced health events prompting them to recognise the importance of screening. Alternatively, there may be additional barriers such as lack of knowledge, inconvenience and fear of medical intervention that cannot be overcome by a simple financial incentive.^20^ However, this does not explain why incentives appear to lead to a decrease in DES attendance.

Incentives may crowd out intrinsic motivation,^21^ but this does not explain the findings, since these patients have not attended appointments for at least 2 years. A possible reason for the negative effect may be that an incentive elicits feelings of dread. The financial incentive may have reinforced an already established perception that screening is unpleasant or painful. Offering payments for tasks leads individuals to report that the experience is more unpleasant when doing the same task for little or no payment,^22 23^ possibly as payment is perceived as recompense for a bad experience. The participants in this trial were a particularly hard-to-reach group, perhaps as they already believe eye screening is an aversive experience. Therefore, they may be prone to interpret incentives as a signal of danger. Alternatively, the voucher may have been perceived as junk mail and ignored.

An observational study in the USA also questions the effectiveness of incentives in DES. Predictors of DES rates were assessed by analysing healthcare claims, and incentives (both provider and patient incentives of $25) were associated with lower screening rates.^24^

There are some limitations and potential sources of bias in this study. Participants were identified through health records that may be incorrect. However, all possible attempts were made to ensure patients included were alive, living at the address listed and eligible for screening. Second, trial participants were invited to a screening centre further from their home than would be the case in the routine screening invite. Yet the impact of these limitations on group comparisons should have been minimal due to randomisation.

The study was powered based on an anticipated 1% attendance in the control group. Observed attendance was close to 8%. Even with the higher-than-anticipated attendance, there is still more than 80% power to detect a 10% increase in attendance. However, given that attendance rates in the incentive groups were lower than in the control group, it seems unlikely that the lack of a positive effect was due to chance alone.

The findings of this London-based study may not be generalisable to other screening programmes in the UK. The population in London is ethnically diverse, with 31% of the Westminster population having a main language that is not English^25^ and a large variation in health. It is therefore possible that many of those in the intervention group did not understand the incentive offer, which may have contributed to the lack of positive findings observed. It is unknown whether the findings generalise to financial incentives in screening programmes in different settings or for other health conditions, given significant variations in methods of screening in different conditions, healthcare system, the population being studied and details of the incentive scheme.

In conclusion, this is a unique randomised controlled trial of the use of financial incentives in a population of patients that have repeatedly not attended DES appointments. The design of the incentives was based on insights from behavioural economics; however, the interventions did not increase screening attendance. There are many factors that predict attendance behaviour, which incentives may not address. Individual financial incentives should be tested in context before widespread adoption that risks financial loss and may possibly adversely affect behaviour.
